# Synthesis, crystal structure and Hirshfeld surface analysis of 4-[3-(4-hy­droxy­phen­yl)-4,5-di­hydro-1*H*-pyrazol-5-yl]-2-meth­oxy­phenol monohydrate

**DOI:** 10.1107/S2056989019013379

**Published:** 2019-10-03

**Authors:** Linh Duong Khanh, My Hanh Trinh Thi, Thuy Quynh Bui Thi, Trung Vu Quoc, Vuong Nguyen Thien, Luc Van Meervelt

**Affiliations:** a Faculty of Chemistry, Hanoi National University of Education, 136 Xuan Thuy, Cau Giay, Hanoi, Vietnam; b Institute for Tropical Technology, Vietnam Academy of Science and Technology, 18 Hoang Quoc Viet, Cau Giay, Hanoi, Vietnam; c Graduate University of Science and Technology, VAST, 18 Hoang Quoc Viet, Cau Giay, Hanoi, Vietnam; d Department of Chemistry, KU Leuven, Biomolecular Architecture, Celestijnenlaan 200F, Leuven (Heverlee), B-3001, Belgium

**Keywords:** crystal structure, pyrazolines, hydrogen bonding, Hirshfeld analysis

## Abstract

In the title pyrazoline derivative, C_16_H_16_N_2_O_3_·H_2_O, the pyrazoline ring has an envelope conformation with the substituted *sp* ^2^ C atom on the flap. The pyrazoline ring makes angles of 86.73 (12) and 13.44 (12)° with the tris­ubstituted and disubstituted benzene rings, respectively. In the crystal structure, the mol­ecules are connected into chains running in the *b*-axis direction by O—H⋯N hydrogen bonding. Parallel chains inter­act through N—H⋯O hydrogen bonds and π–π stacking of the tris­ubstituted phenyl rings. The major contribution to the surface contacts are H⋯H contacts (44.3%) as concluded from a Hirshfeld surface analysis.

## Abstract 2

In the title pyrazoline derivative, the pyrazoline ring makes angles of 86.73 (12) and 13.44 (12)° with the tris­ubstituted and disubstituted benzene rings, respectively. In the crystal structure, the mol­ecules are connected into chains running in the *b*-axis direction by O—H⋯N hydrogen bonding. Parallel chains inter­act through N—H⋯O hydrogen bonds and π–π stacking of the tris­ubstituted phenyl rings.

## Chemical context   

Chalcones are one of the most important classes of flavonoids. Natural and synthetic chalcone derivatives have shown a variety of promising biological activities such as anti-inflammatory, anti-gout, anti-histaminic, anti-oxidant, anti-obesity, anti-protozoal, hypnotic and anti-spasmodic activities (Gomes *et al.*, 2017 ▸). Heterocyclic compounds including pyrazolines can be synthesized from chalcone derivatives. Many compounds containing pyrazolines show different biological activities and are known to act as anti­cancer (Johnson *et al.*, 2007 ▸; Gomha *et al.*, 2017 ▸), anti­microbial (Patel *et al.*, 2016 ▸), anti­tubercular (Taj *et al.*, 2011 ▸), anti-inflammatory (Malhotra *et al.*, 2010 ▸), anti­convulsant (Siddiqui *et al.*, 2009 ▸), anti-amoebic (Bhat *et al.*, 2009 ▸), anti­oxidant (Srinivasan *et al.*, 2007 ▸), anti­viral (Gomha *et al.*, 2016 ▸), anti­bacterial **(**Kumar *et al.*, 2008 ▸) and anti­nociceptive (Kaplancikli *et al.*, 2009 ▸) agents.

Pyrazoline derivatives have been synthesized by condensation of chalcones with hydrazine derivatives using conventional synthesis (Shahare *et al.*, 2009 ▸; Sridhar *et al.*, 2012 ▸) and microwave-assisted synthesis (Kumar *et al.*, 2008 ▸; Patel *et al.*, 2016 ▸).

In this article, we report the synthesis of a chalcone derivative by condensation of vanillin with *p*-hy­droxy­aceto­phenone and subsequent cyclization of this chalcone by reaction with hydrazine hydrate. Furthermore the mol­ecular and crystal structure of the title compound, **2**, are presented together with a Hirshfeld surface analysis and non-covalent inter­action plots.
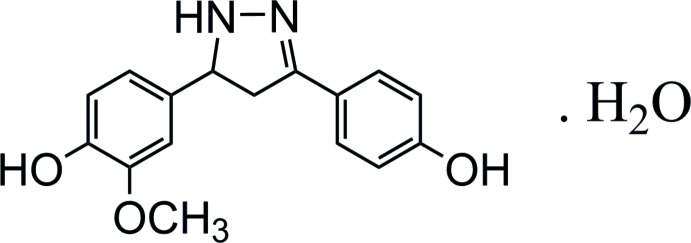


## Structural commentary   

**Figure 1:**
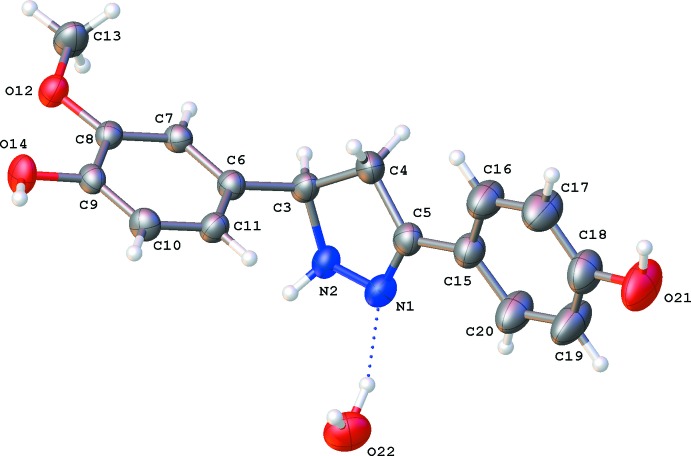
A view of the mol­ecular structure of **2**, with atom labels and displacement ellipsoids drawn at the 50% probability level. H atoms are shown as small circles of arbitrary radii and the O—H⋯N inter­action as a dotted blue line.

The title compound crystallizes in the ortho­rhom­bic space group *Pbca* with one mol­ecule and a water mol­ecule in the asymmetric unit (Fig. 1 ▸). The pyrazoline ring (N1/N2/C3–C5; r.m.s. deviation = 0.078 Å) is slightly twisted on N2—C3 [puckering parameters: *Q*(2) = 0.175 (2) Å, Φ(2) = 60.7 (7)°]. There is a clear difference in both C—N bond distances in the pyrazoline ring: N1=C5 shows double-bond character [1.287 (3) Å] while N2—C3 [1.496 (3) Å] is a single bond. The dihedral angle between the two benzene rings is 80.66 (11)°. The planes of the C6–C11 benzene ring (r.m.s. deviation = 0.004 Å) and the pyrazoline ring make an angle of 86.73 (12)°. For the C15–C20 benzene ring (r.m.s. deviation = 0.006 Å), the dihedral angle with the pyrazoline ring is only 13.44 (12)°. Both the hy­droxy and meth­oxy substituents of the C6–C11 phenyl group are within the phenyl plane with deviations of 0.011 (1) (O12), 0.166 (2) (C13) and −0.057 (2) Å (O14).

## Supra­molecular features and Hirshfeld surface analysis   

**Figure 2:**
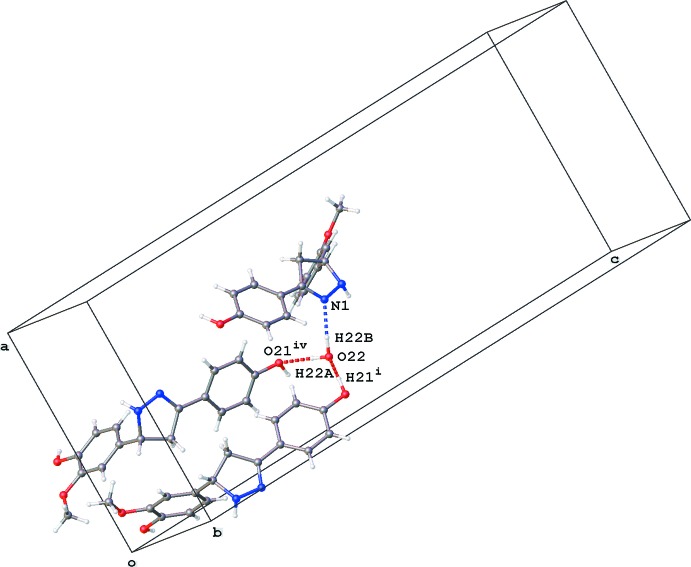
Partial crystal packing of **2**, showing the inter­actions of water mol­ecule O22. O—H⋯N and O—H⋯O inter­actions are shown as blue and red dashed lines, respectively (see Table 1 ▸ for symmetry codes).

**Figure 3:**
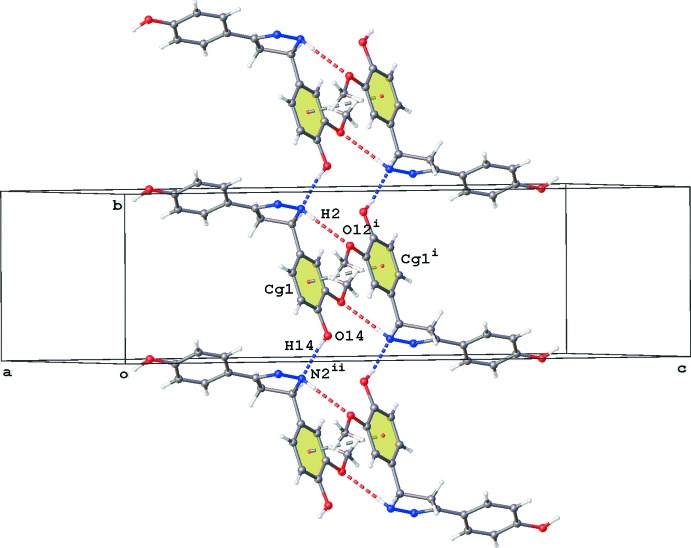
Partial crystal packing of **2**, showing the chain formation along the *b* axis by O—H⋯N inter­actions (blue dashed lines). Parallel chains are linked by N—H⋯O (red dashed lines) and π–π inter­actions (grey dashed lines; *Cg*1 is the centroid of the C6–C11 ring; see Table 1 ▸ for symmetry codes).

In the crystal of **2**, the O22 water mol­ecule bridges three mol­ecules by O—H⋯N and O—H⋯O hydrogen-bonding inter­actions with the N1 atom and the O21-hy­droxy group (Fig. 2 ▸, Table 1 ▸). The pyrazoline N2 atom acts as a hydrogen-bond acceptor to the second O14-hy­droxy group, resulting in chain formation along the *b*-axis direction (Fig. 3 ▸, Table 1 ▸). Parallel chains linked by inversion inter­act in two different ways. First, the N2 hydrogen atom acts also as hydrogen-bond donor to the O12-meth­oxy group. In addition, both chains inter­act by π–π stacking [*Cg*1⋯*Cg*1(−*x* + 1, −*y* + 1, −*z* + 1) = 3.6627 (11) Å; slippage 1.442 Å; *Cg*1 is the centroid of the C6–C11 ring]. In addition, a C—H⋯O inter­action is observed in the crystal packing (Table 1 ▸). No voids are observed in the crystal packing of **2**.

**Figure 4:**
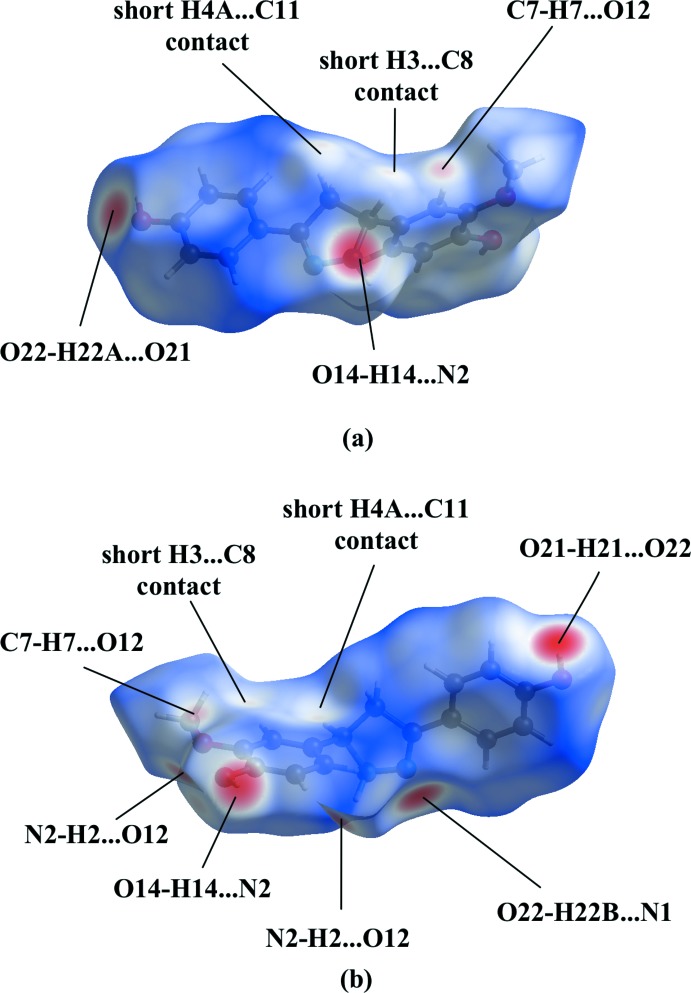
Two views of the Hirshfeld surface mapped over *d*_norm_ for **2** in the range −0.7348 to +1.5269 arbitrary units.

**Figure 5:**
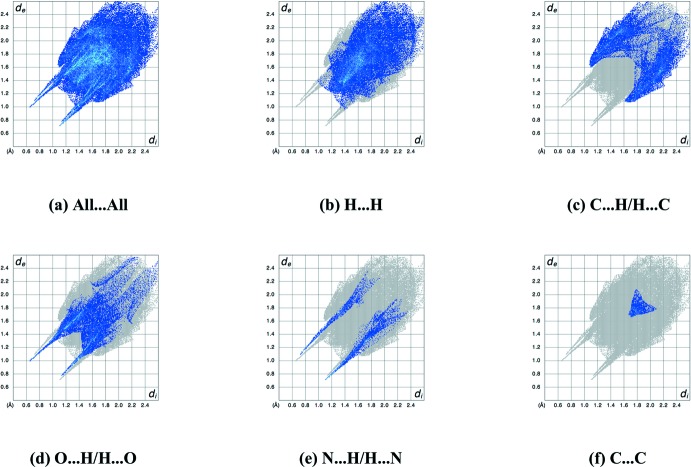
Full two-dimensional fingerprint plots for **2**, showing (*a*) all inter­actions, and delineated into (*b*) H⋯H, (*c*) C⋯H/H⋯C, (*d*) O⋯H/H⋯O, (*e*) N⋯H/H⋯N and (*f*) C⋯C inter­actions. The *d*_i_ and *d*_e_ values are the closest inter­nal and external distances (in Å) from a given point on the Hirshfeld surface.

The Hirshfeld surface (calculated using *CrystalExplorer*; Turner *et al.*, 2017 ▸) mapped over *d*_norm_ in Fig. 4 ▸ also gives the usual indications of these inter­molecular inter­actions through the appearance of bright-red spots near participating atoms (Spackman & Jayatilaka, 2009 ▸). In addition to the inter­actions already discussed, faint-red spots near atoms C8, C11, H3 and H4*A* illustrate short C⋯H contacts (H4*A*⋯C11 = 2.83 Å, H3⋯C8 = 2.80 Å). The associated two-dimensional fingerprint plots (McKinnon *et al.*, 2007 ▸) were used to further explore the inter­molecular contacts (Fig. 5 ▸) and indicate that the major contribution is from H⋯H contacts, corresponding to 44.3% of the fingerprint plot (Fig. 5 ▸ *b*) followed by recip­rocal C⋯H/H⋯C contacts (25.1%, Fig. 5 ▸ *c*). Significant contributions come from reciprocal O⋯H/H⋯O (20.7%) and N⋯H/H⋯N (7.0%) contacts, which appear as two symmetrical spikes at *d*_e_ + *d*_i_ = 1.65 and 1.80 Å, respectively (Fig. 5 ▸ *d*,*e*). A further small contribution is from C⋯C contacts (2.3%, Fig. 5 ▸ *f*).

Based on the Hirshfeld surface analysis, enrichment ratios (ER, Table 2 ▸) were calculated by comparing the contacts in the crystal with those computed as if all types of contact have the same probability of forming (Jelsch *et al.*, 2014 ▸). A ratio greater than unity for a pair of elements indicates a high likelihood of forming contacts in the crystal. This is the case for N⋯H and O⋯H contacts, which is consistent with the high propensity for the formation of O—H⋯N and O/N/C—H⋯O hydrogen bonds. C⋯H contacts are enriched because of the presence of aromatic rings, H⋯H contacts are found to have the usual enrichment ratios slightly lower than unity.

## Database survey   

A search of the Cambridge Structural Database (CSD, Version 5.40, update of May 2019; Groom *et al.*, 2016 ▸) for 2-pyrazoline derivatives gave 134 hits, of which 37 are 3,5-disubstituted (only organic mol­ecules were considered). Where both substituents on the pyrazoline ring are aromatic rings, three 2-pyrazoline derivatives were found with substituted benzene rings at position 3 and a 2-naphthyl ring system at position 5. In addition, two structures have substituted benzene rings as both substituents and are very similar to **2**. The first one, 2-meth­oxy-4-[3-(3-nitro­phen­yl)-4,5-di­hydro-1*H*-pyrazol-5-yl]phenol (refcode UJUDOU; Inturi *et al.*, 2016 ▸), crystallizes in *P*2_1_/c with one mol­ecule in the asymmetric unit. The pyrazoline ring has an envelope conformation with the substituted *sp* ^2^ C atom on the flap. The dihedral angle between the phenyl rings is 49.37 (8)°, that between the pyrazoline ring and the nitro­phenyl ring is 9.7 (1)° and that between the pyrazoline ring and the meth­oxy­phenol ring is 56. 78 (9)°. The second structure, 3-(2′-hy­droxy-5′-meth­oxy-phen­yl)-5-(3-meth­oxy-4-hy­droxy­phen­yl)-4,5-di­hydro-1*H*-pyrazole (RES­JUV; Gupta *et al.*, 2006 ▸), crystallizes in *P*bca with one mol­ecule in the asymmetric unit. The conformation of the pyrazoline ring is the same as that in UJUDOU. The phenyl rings make an angle of 56.0 (1)°, while the dihedral angles between the pyrazoline ring and the phenyl rings at atom C3 and C5 are 12.1 (1) and 68.2 (1)°, respectively.

## Synthesis and crystallization   

**Figure 6:**
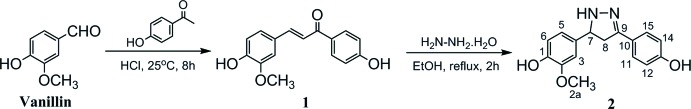
Reaction scheme for the synthesis of compound **2**.

The reaction scheme for the synthesis of **2** starting from vanillin is given in Fig. 6 ▸. (*E*)-3-(4-Hy­droxy-3-meth­oxy­phen­yl)-1-(4-hy­droxy­phen­yl)prop-2-en-1-one, **1**, was synthesized as described in a previous study (Duong Khanh *et al.*, 2018 ▸).

***Synthesis of 4-(3-(4-hy­droxy­phen­yl)-4,5-di­hydro-1H-pyrazol-5-yl)-2-meth­oxy­phenol (2):***

A mixture of chalcone **1** (0.01 mol), 2.5 mL of hydrazine hydrate and 25 mL of ethanol was refluxed at 353 K for 2 h. After pouring the reaction mixture into 200 mL of ice–water, the crude solid product was isolated by vacuum filtration, washed several times with cold water and recrystallized from ethanol:water (1:2) to give yellow crystals (2.24 g, yield 79%), m.p. 465 K. ^1^H NMR [Bruker XL-500, 500 MHz, *d*_6_-DMSO, δ (ppm), *J* (Hz), see Fig. 6 ▸ for numbering scheme]: 6.71 (*d*, 1H, *J* = 8.0, H2); 3.75 (*s*, 3H, H2a); 6.74 (*d*, 1H, *J* = 1.5, H3); 6.95 (*d*, 1H, *J* = 1.5, H5), 4.67 (*t*, 1H, H7); 3.30 (*dd*, 1H, *J* = 11.0; 16.0, H8a); 2.76 (*dd*, 1H, *J* = 11.0, 16.0, H8b), 7.45 (*d*, 2H, *J* = 8.5, H11 and H15); 6.76 (*d*, 2H, *J* = 8.5, H12 and H14).

## Refinement   

Crystal data, data collection and structure refinement details are summarized in Table 3 ▸. The O- and N-bound H atoms H2, H14, H21, H22*A* and H22*B* were found in difference electron density maps and refined freely. The other H atoms were placed in idealized positions and included as riding contributions with *U*_iso_(H) values of 1.2*U*_eq_ or 1.5*U*_eq_ of the parent atoms, with C—H distances of 0.93 (aromatic), 0.98 (CH), 0.97 (CH_2_) and 0.96 Å (CH_3_). In the final cycles of refinement, eight outliers were omitted.

## Supplementary Material

Crystal structure: contains datablock(s) I. DOI: 10.1107/S2056989019013379/sj5579sup1.cif

Structure factors: contains datablock(s) I. DOI: 10.1107/S2056989019013379/sj5579Isup2.hkl

Supporting information file. DOI: 10.1107/S2056989019013379/sj5579Isup3.cml

CCDC references: 1956698, 1956698

Additional supporting information:  crystallographic information; 3D view; checkCIF report

## Figures and Tables

**Table 1:** Hydrogen-bond geometry (Å, °)

*D*—H⋯*A*	*D*—H	H⋯*A*	*D*⋯*A*	*D*—H⋯*A*
N2—H2⋯O12^i^	0.87 (2)	2.36 (2)	3.209 (2)	167 (2)
O14—H14⋯N2^ii^	0.91 (3)	1.89 (3)	2.760 (2)	158 (3)
O21—H21⋯O22^iii^	0.98 (4)	1.66 (4)	2.633 (3)	169 (4)
O22—H22*A*⋯O21^iv^	0.91 (4)	1.99 (4)	2.891 (3)	171 (3)
O22—H22*B*⋯N1	0.98 (4)	1.84 (4)	2.794 (3)	166 (3)
C7—H7⋯O12^v^	0.93	2.56	3.465 (2)	165

Symmetry codes: (i) 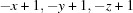; (ii) 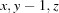; (iii) 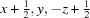; (iv) 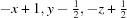; (v) 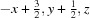.

**Table 2:** Enrichment ratios for **2**

Parameter	Ratio
H⋯H	0.89
C⋯H	1.18
O⋯H	1.39
N⋯H	1.41
C⋯C	1.02

**Table 3:** Experimental details

Crystal data	
Chemical formula	C_16_H_16_N_2_O_3_·H_2_O
*M*_r_	302.32
Crystal system, space group	Orthorhombic, *P* *b* *c* *a*
Temperature (K)	293
*a*, *b*, *c* (Å)	12.1452 (5), 8.1784 (3), 31.2738 (12)
*V* (Å^3^)	3106.4 (2)
*Z*	8
Radiation type	Mo *K*α
μ (mm^−1^)	0.09
Crystal size (mm)	0.5 × 0.2 × 0.05

Data collection	
Diffractometer	Rigaku Oxford Diffraction SuperNova, Single source at offset/far, Eos
Absorption correction	Multi-scan (*CrysAlis PRO*; Rigaku OD, 2018 ▸)
*T*_min_, *T*_max_	0.697, 1.000
No. of measured, independent and observed [*I* > 2σ(*I*)] reflections	16817, 3172, 2241
*R*_int_	0.039
(sin θ/λ)_max_ (Å^−1^)	0.625

Refinement	
*R*[*F* ^2^ > 2σ(*F* ^2^)], *wR*(*F* ^2^), *S*	0.050, 0.115, 1.03
No. of reflections	3172
No. of parameters	220
H-atom treatment	H atoms treated by a mixture of independent and constrained refinement
Δρ_max_, Δρ_min_ (e Å^−3^)	0.18, −0.19

Computer programs: *CrysAlis PRO* (Rigaku OD, 2018 ▸), *SHELXT2014* (Sheldrick, 2015*a* ▸), *SHELXL2016* (Sheldrick, 2015*b* ▸) and *OLEX2* (Dolomanov *et al.*, 2009 ▸).

## supplementary crystallographic information

### Crystal data

**Table:**

C_16_H_16_N_2_O_3_·H_2_O	*D*_x_ = 1.293 Mg m^−^^3^
*M**_r_* = 302.32	Mo *K*α radiation, λ = 0.71073 Å
Orthorhombic, *P**b**c**a*	Cell parameters from 4406 reflections
*a* = 12.1452 (5) Å	θ = 3.1–24.8°
*b* = 8.1784 (3) Å	µ = 0.09 mm^−^^1^
*c* = 31.2738 (12) Å	*T* = 293 K
*V* = 3106.4 (2) Å^3^	Plate, yellow
*Z* = 8	0.5 × 0.2 × 0.05 mm
*F*(000) = 1280	

### Data collection

**Table:**

Rigaku Oxford Diffraction SuperNova, Single source at offset/far, Eos diffractometer	3172 independent reflections
Radiation source: micro-focus sealed X-ray tube, SuperNova (Mo) X-ray Source	2241 reflections with *I* > 2σ(*I*)
Mirror monochromator	*R*_int_ = 0.039
Detector resolution: 15.9631 pixels mm^-1^	θ_max_ = 26.4°, θ_min_ = 2.6°
ω scans	*h* = −15→15
Absorption correction: multi-scan (CrysAlis PRO; Rigaku OD, 2018)	*k* = −10→9
*T*_min_ = 0.697, *T*_max_ = 1.000	*l* = −38→39
16817 measured reflections	

### Refinement

**Table:**

Refinement on *F*^2^	0 restraints
Least-squares matrix: full	Hydrogen site location: mixed
*R*[*F*^2^ > 2σ(*F*^2^)] = 0.050	H atoms treated by a mixture of independent and constrained refinement
*wR*(*F*^2^) = 0.115	*w* = 1/[σ^2^(*F*_o_^2^) + (0.0321*P*)^2^ + 1.8202*P*] where *P* = (*F*_o_^2^ + 2*F*_c_^2^)/3
*S* = 1.03	(Δ/σ)_max_ < 0.001
3172 reflections	Δρ_max_ = 0.18 e Å^−^^3^
220 parameters	Δρ_min_ = −0.19 e Å^−^^3^

### Special details

**Table:**

Geometry. All esds (except the esd in the dihedral angle between two l.s. planes) are estimated using the full covariance matrix. The cell esds are taken into account individually in the estimation of esds in distances, angles and torsion angles; correlations between esds in cell parameters are only used when they are defined by crystal symmetry. An approximate (isotropic) treatment of cell esds is used for estimating esds involving l.s. planes.

### Fractional atomic coordinates and isotropic or equivalent isotropic displacement parameters (Å^2^)

**Table:**

	*x*	*y*	*z*	*U*_iso_*/*U*_eq_	
O12	0.67375 (11)	0.32986 (16)	0.53007 (4)	0.0374 (4)	
O14	0.56063 (13)	0.13497 (17)	0.48138 (5)	0.0433 (4)	
N2	0.51402 (15)	0.8855 (2)	0.42476 (5)	0.0347 (4)	
N1	0.49999 (14)	0.9151 (2)	0.38046 (5)	0.0384 (4)	
O22	0.29208 (18)	0.8202 (3)	0.35137 (6)	0.0677 (5)	
O21	0.64259 (19)	1.0090 (3)	0.18438 (6)	0.0803 (7)	
C8	0.63357 (15)	0.3973 (2)	0.49290 (6)	0.0287 (4)	
C9	0.57465 (16)	0.2905 (2)	0.46661 (6)	0.0306 (4)	
C7	0.64750 (15)	0.5586 (2)	0.48084 (6)	0.0298 (4)	
H7	0.686502	0.629245	0.498546	0.036*	
C6	0.60392 (16)	0.6168 (2)	0.44254 (6)	0.0312 (4)	
C11	0.54679 (16)	0.5094 (2)	0.41631 (6)	0.0346 (5)	
H11	0.517910	0.546425	0.390526	0.042*	
C3	0.61976 (16)	0.7953 (2)	0.43154 (7)	0.0348 (5)	
H3	0.660239	0.848056	0.454879	0.042*	
C10	0.53234 (17)	0.3472 (2)	0.42823 (6)	0.0354 (5)	
H10	0.494061	0.276326	0.410349	0.042*	
C5	0.59163 (17)	0.8914 (2)	0.36073 (7)	0.0376 (5)	
C15	0.60588 (19)	0.9252 (3)	0.31515 (7)	0.0431 (5)	
C4	0.68056 (18)	0.8284 (3)	0.38953 (7)	0.0450 (6)	
H4A	0.738080	0.909351	0.393418	0.054*	
H4B	0.712858	0.728975	0.378196	0.054*	
C13	0.7444 (2)	0.4300 (3)	0.55573 (7)	0.0482 (6)	
H13A	0.803642	0.471013	0.538454	0.072*	
H13B	0.773932	0.366112	0.578791	0.072*	
H13C	0.703076	0.520021	0.567146	0.072*	
C16	0.6949 (2)	0.8613 (3)	0.29308 (8)	0.0574 (7)	
H16	0.746563	0.798987	0.307775	0.069*	
C18	0.6333 (2)	0.9799 (3)	0.22753 (8)	0.0597 (7)	
C20	0.5313 (2)	1.0207 (3)	0.29215 (8)	0.0615 (7)	
H20	0.471444	1.067128	0.306189	0.074*	
C17	0.7088 (2)	0.8877 (3)	0.24971 (8)	0.0654 (8)	
H17	0.769099	0.843147	0.235584	0.079*	
C19	0.5450 (2)	1.0475 (4)	0.24884 (8)	0.0723 (9)	
H19	0.494359	1.111272	0.234017	0.087*	
H2	0.4564 (18)	0.832 (3)	0.4332 (7)	0.042 (6)*	
H22A	0.317 (3)	0.729 (5)	0.3380 (11)	0.109 (14)*	
H22B	0.364 (3)	0.867 (4)	0.3581 (10)	0.106 (12)*	
H14	0.533 (2)	0.072 (4)	0.4599 (9)	0.082 (10)*	
H21	0.705 (3)	0.949 (5)	0.1719 (12)	0.130 (14)*	

### Atomic displacement parameters (Å^2^)

**Table:**

	*U*^11^	*U*^22^	*U*^33^	*U*^12^	*U*^13^	*U*^23^
O12	0.0429 (8)	0.0318 (7)	0.0375 (8)	−0.0015 (6)	−0.0074 (6)	0.0036 (6)
O14	0.0619 (10)	0.0250 (8)	0.0429 (9)	−0.0082 (7)	−0.0049 (8)	0.0019 (7)
N2	0.0417 (10)	0.0297 (9)	0.0329 (9)	0.0003 (8)	−0.0026 (8)	0.0031 (8)
N1	0.0431 (10)	0.0380 (10)	0.0340 (9)	0.0041 (8)	−0.0045 (8)	0.0021 (8)
O22	0.0638 (13)	0.0794 (14)	0.0599 (12)	0.0010 (11)	−0.0157 (10)	−0.0056 (11)
O21	0.0997 (17)	0.0965 (16)	0.0446 (11)	0.0282 (13)	0.0183 (11)	0.0222 (11)
C8	0.0293 (10)	0.0280 (10)	0.0289 (10)	0.0025 (8)	0.0020 (8)	0.0011 (8)
C9	0.0316 (10)	0.0238 (9)	0.0365 (11)	0.0002 (8)	0.0038 (9)	0.0004 (8)
C7	0.0300 (10)	0.0264 (10)	0.0331 (11)	−0.0013 (8)	−0.0015 (8)	−0.0024 (8)
C6	0.0329 (10)	0.0271 (10)	0.0336 (11)	−0.0006 (8)	0.0003 (8)	0.0001 (9)
C11	0.0368 (11)	0.0342 (11)	0.0329 (11)	0.0002 (9)	−0.0044 (9)	0.0028 (9)
C3	0.0360 (11)	0.0297 (10)	0.0387 (12)	−0.0042 (8)	−0.0078 (9)	0.0049 (9)
C10	0.0400 (11)	0.0308 (11)	0.0353 (11)	−0.0049 (9)	−0.0016 (9)	−0.0054 (9)
C5	0.0411 (12)	0.0316 (11)	0.0402 (12)	−0.0015 (9)	−0.0009 (10)	0.0040 (9)
C15	0.0464 (13)	0.0430 (12)	0.0400 (12)	0.0030 (10)	0.0018 (10)	0.0069 (10)
C4	0.0384 (12)	0.0435 (12)	0.0531 (14)	−0.0057 (9)	−0.0008 (10)	0.0162 (11)
C13	0.0535 (14)	0.0481 (13)	0.0430 (13)	−0.0083 (11)	−0.0160 (11)	0.0040 (11)
C16	0.0575 (16)	0.0672 (17)	0.0476 (14)	0.0172 (13)	0.0071 (12)	0.0136 (13)
C18	0.0713 (18)	0.0653 (17)	0.0426 (14)	0.0109 (14)	0.0114 (13)	0.0133 (13)
C20	0.0671 (17)	0.0702 (18)	0.0473 (14)	0.0255 (14)	0.0129 (13)	0.0154 (13)
C17	0.0643 (17)	0.0809 (19)	0.0511 (15)	0.0235 (15)	0.0170 (13)	0.0109 (15)
C19	0.079 (2)	0.089 (2)	0.0490 (15)	0.0365 (17)	0.0091 (14)	0.0240 (15)

### Geometric parameters (Å, º)

**Table:**

O12—C8	1.376 (2)	C3—H3	0.9800
O12—C13	1.432 (2)	C3—C4	1.531 (3)
O14—C9	1.364 (2)	C10—H10	0.9300
O14—H14	0.91 (3)	C5—C15	1.462 (3)
N2—N1	1.417 (2)	C5—C4	1.498 (3)
N2—C3	1.496 (3)	C15—C16	1.385 (3)
N2—H2	0.87 (2)	C15—C20	1.396 (3)
N1—C5	1.287 (3)	C4—H4A	0.9700
O22—H22A	0.91 (4)	C4—H4B	0.9700
O22—H22B	0.98 (4)	C13—H13A	0.9600
O21—C18	1.375 (3)	C13—H13B	0.9600
O21—H21	0.98 (4)	C13—H13C	0.9600
C8—C9	1.397 (3)	C16—H16	0.9300
C8—C7	1.383 (3)	C16—C17	1.384 (3)
C9—C10	1.386 (3)	C18—C17	1.375 (3)
C7—H7	0.9300	C18—C19	1.379 (3)
C7—C6	1.393 (3)	C20—H20	0.9300
C6—C11	1.387 (3)	C20—C19	1.382 (3)
C6—C3	1.513 (3)	C17—H17	0.9300
C11—H11	0.9300	C19—H19	0.9300
C11—C10	1.389 (3)		
			
C8—O12—C13	117.18 (15)	N1—C5—C4	112.78 (18)
C9—O14—H14	108.7 (18)	C15—C5—C4	124.46 (19)
N1—N2—C3	109.04 (16)	C16—C15—C5	120.5 (2)
N1—N2—H2	106.6 (14)	C16—C15—C20	117.4 (2)
C3—N2—H2	113.6 (14)	C20—C15—C5	122.1 (2)
C5—N1—N2	109.81 (17)	C3—C4—H4A	111.1
H22A—O22—H22B	97 (3)	C3—C4—H4B	111.1
C18—O21—H21	112 (2)	C5—C4—C3	103.23 (17)
O12—C8—C9	115.37 (16)	C5—C4—H4A	111.1
O12—C8—C7	124.70 (17)	C5—C4—H4B	111.1
C7—C8—C9	119.93 (17)	H4A—C4—H4B	109.1
O14—C9—C8	116.60 (17)	O12—C13—H13A	109.5
O14—C9—C10	124.02 (18)	O12—C13—H13B	109.5
C10—C9—C8	119.36 (17)	O12—C13—H13C	109.5
C8—C7—H7	119.5	H13A—C13—H13B	109.5
C8—C7—C6	120.92 (18)	H13A—C13—H13C	109.5
C6—C7—H7	119.5	H13B—C13—H13C	109.5
C7—C6—C3	118.49 (17)	C15—C16—H16	119.2
C11—C6—C7	118.83 (17)	C17—C16—C15	121.7 (2)
C11—C6—C3	122.68 (17)	C17—C16—H16	119.2
C6—C11—H11	119.7	O21—C18—C17	122.4 (2)
C6—C11—C10	120.59 (18)	O21—C18—C19	118.0 (2)
C10—C11—H11	119.7	C17—C18—C19	119.7 (2)
N2—C3—C6	113.52 (16)	C15—C20—H20	119.5
N2—C3—H3	108.6	C19—C20—C15	121.0 (2)
N2—C3—C4	101.83 (15)	C19—C20—H20	119.5
C6—C3—H3	108.6	C16—C17—H17	120.0
C6—C3—C4	115.28 (17)	C18—C17—C16	119.9 (2)
C4—C3—H3	108.6	C18—C17—H17	120.0
C9—C10—C11	120.36 (18)	C18—C19—C20	120.3 (2)
C9—C10—H10	119.8	C18—C19—H19	119.9
C11—C10—H10	119.8	C20—C19—H19	119.9
N1—C5—C15	122.76 (19)		
			
O12—C8—C9—O14	2.2 (2)	C7—C6—C3—N2	−121.91 (19)
O12—C8—C9—C10	−179.16 (17)	C7—C6—C3—C4	121.2 (2)
O12—C8—C7—C6	180.00 (17)	C6—C11—C10—C9	0.2 (3)
O14—C9—C10—C11	177.47 (18)	C6—C3—C4—C5	108.50 (19)
N2—N1—C5—C15	−174.82 (18)	C11—C6—C3—N2	57.3 (3)
N2—N1—C5—C4	4.4 (2)	C11—C6—C3—C4	−59.6 (3)
N2—C3—C4—C5	−14.9 (2)	C3—N2—N1—C5	−15.0 (2)
N1—N2—C3—C6	−106.21 (19)	C3—C6—C11—C10	−178.59 (18)
N1—N2—C3—C4	18.3 (2)	C5—C15—C16—C17	178.3 (2)
N1—C5—C15—C16	−163.9 (2)	C5—C15—C20—C19	−178.3 (3)
N1—C5—C15—C20	15.7 (3)	C15—C5—C4—C3	−173.42 (19)
N1—C5—C4—C3	7.3 (2)	C15—C16—C17—C18	0.2 (4)
O21—C18—C17—C16	−179.5 (3)	C15—C20—C19—C18	−0.1 (5)
O21—C18—C19—C20	179.4 (3)	C4—C5—C15—C16	17.0 (3)
C8—C9—C10—C11	−1.1 (3)	C4—C5—C15—C20	−163.5 (2)
C8—C7—C6—C11	−0.6 (3)	C13—O12—C8—C9	173.54 (18)
C8—C7—C6—C3	178.69 (17)	C13—O12—C8—C7	−6.8 (3)
C9—C8—C7—C6	−0.3 (3)	C16—C15—C20—C19	1.2 (4)
C7—C8—C9—O14	−177.51 (17)	C20—C15—C16—C17	−1.3 (4)
C7—C8—C9—C10	1.1 (3)	C17—C18—C19—C20	−1.0 (5)
C7—C6—C11—C10	0.6 (3)	C19—C18—C17—C16	0.9 (5)

### Hydrogen-bond geometry (Å, º)

**Table:**

*D*—H···*A*	*D*—H	H···*A*	*D*···*A*	*D*—H···*A*
N2—H2···O12^i^	0.87 (2)	2.36 (2)	3.209 (2)	167 (2)
O14—H14···N2^ii^	0.91 (3)	1.89 (3)	2.760 (2)	158 (3)
O21—H21···O22^iii^	0.98 (4)	1.66 (4)	2.633 (3)	169 (4)
O22—H22*A*···O21^iv^	0.91 (4)	1.99 (4)	2.891 (3)	171 (3)
O22—H22*B*···N1	0.98 (4)	1.84 (4)	2.794 (3)	166 (3)
C7—H7···O12^v^	0.93	2.56	3.465 (2)	165

Symmetry codes: (i) −*x*+1, −*y*+1, −*z*+1; (ii) *x*, *y*−1, *z*; (iii) *x*+1/2, *y*, −*z*+1/2; (iv) −*x*+1, *y*−1/2, −*z*+1/2; (v) −*x*+3/2, *y*+1/2, *z*.
